# Chromium(II)-isophthalate 2D MOF with Redox-Tailorable Gas Adsorption Selectivity

**DOI:** 10.1021/acsami.4c06228

**Published:** 2024-08-19

**Authors:** Michał K. Leszczyński, Katarzyna Niepiekło, Michał Terlecki, Iwona Justyniak, Janusz Lewiński

**Affiliations:** † Faculty of Chemistry, Warsaw University of Technology, Noakowskiego 3, 00-664 Warsaw, Poland; ‡ Institute of Physical Chemistry, Polish Academy of Sciences, Kasprzaka 44/52, 01-224 Warsaw, Poland

**Keywords:** metal−organic frameworks, chromium, redox, postsynthetic modification, molecular sieve, two-dimensional materials

## Abstract

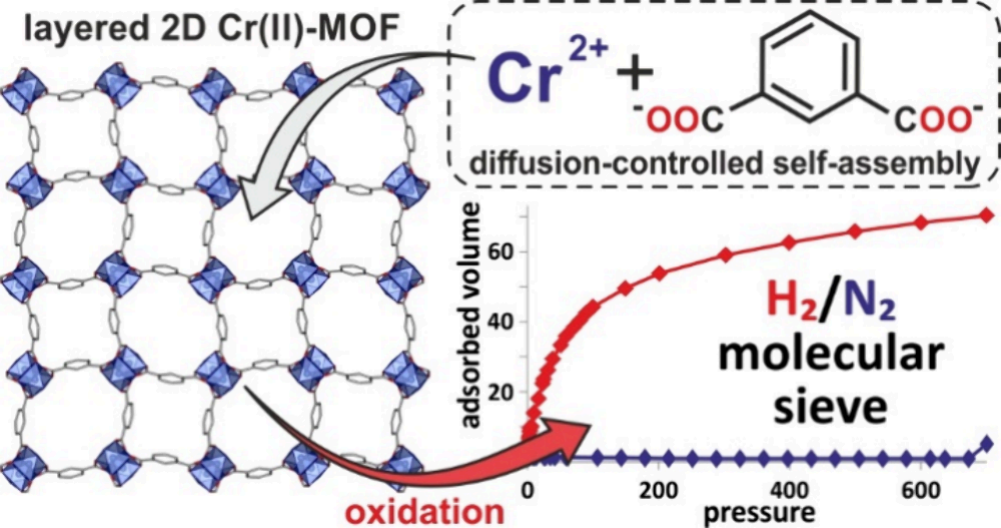

Redox-active metal–organic frameworks (MOFs) are very promising materials due to their potential capabilities for postsynthetic modification aimed at tailoring their application properties. However, the research field related to redox-active MOFs is still relatively underdeveloped, which limits their practical application. We investigated the self-assembly process of Cr(II) ions and isophthalate (m-bdc) linkers, which have been previously demonstrated to yield 0D metal–organic polyhedra. However, using the diffusion-controlled synthetic approach, we demonstrate the selective preparation of a 2D-layered Cr(II)-based MOF material [Cr(m-bdc)]·H_2_O (**1·H**_**2**_**O**). Remarkably, the controlled oxidation of the developed 2D MOF using nitric oxide or dry oxygen resulted in modified porous materials with excellent H_2_/N_2_ adsorption selectivities.

## Introduction

One of the key application areas of metal–organic frameworks (MOFs) involves selective gas adsorption aimed at separation of gas mixtures. To this end, ultramicroporous (pore apertures <0.7 nm) MOFs appear as promising materials for gas storage and separation due to very specific internal pore geometries often allowing for distinctive interactions with various guest molecules.^1,2^ Over the last 2 decades, application of MOFs as hydrogen adsorption materials has been attracting increasing researchers’ attention.^3−5^ Concomitantly, efficient separation of H_2_/N_2_ mixtures is highly desired with regard to energy-related applications, which has sparked numerous experimental and theoretical studies in this area, mostly focused on MOF-based membranes.^6−9^ Alternatively, flexible MOFs appear as promising materials for H_2_ separation based on selective adsorption due to possible structural transformations triggered by the presence of guest molecules.^7^ The growing interest in flexible MOFs is reflected by increasing attention devoted to layered 2D MOFs, with enhanced flexibility and the ability to adopt various stacking modes, providing promising avenues for the design of modern functional materials.^10−13^

**Figure 1:**
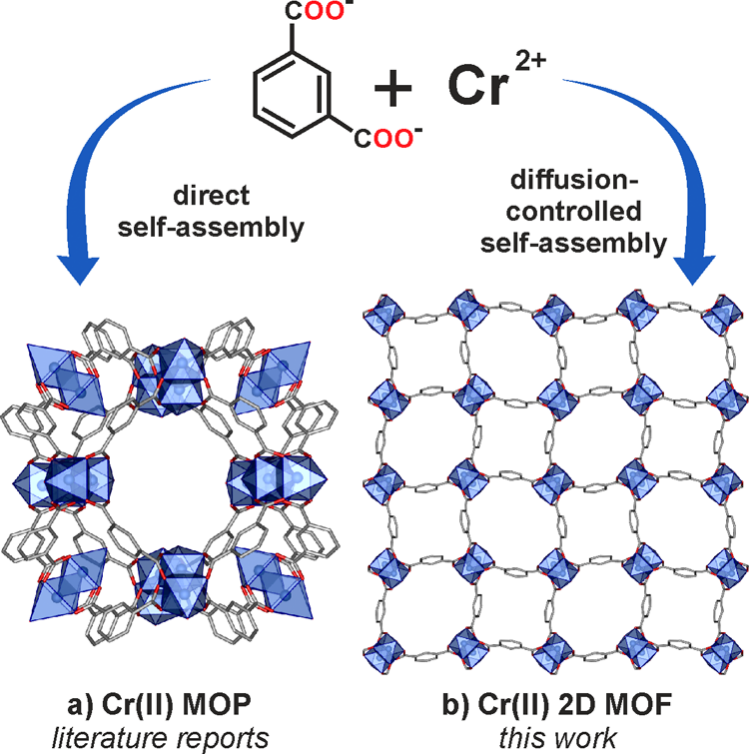
Overview of the products resulting from the self-assembly of Cr^2+^ cations and isophthalate linkers: (a) MOP reported by Zhou and co-workers.^25^ (b) 2D MOF reported here.

Another research direction aimed at the development of cutting-edge functional materials involves harnessing their chemical reactivity for rational functionalization. For example, redox-active MOFs appear very promising with regard to modern applications such as catalysis, energy storage, selective gas adsorption, or sensors, but this research area is still vastly underdeveloped.^14,15^ In particular, MOFs involving redox-active metal centers are considerably rare, but several examples involving, e.g., Ti, V, Cr, Mn, Fe, Co, Ni, Cu, and Ce-based systems have been published as promising materials for modern applications.^15^ Chromium(II)-based MOFs, first prepared by Long and co-workers,^16^ have shown some exciting application potential,^17,18^ but over the past decade, the progress in this area has been limited to only a handful of papers reporting on new Cr(II)-based MOFs, which is likely related to significant practical challenge in the synthesis and handling of these air-sensitive materials.^19−22^ The limited scale of scientific progress in this area is well demonstrated by comparison of Cr(II) and Cu(II)-based systems, which form isostructural molecular paddlewheel-type carboxylate units, yet the latter is represented by thousands of coordination polymer structures published so far.^23^ Apart from the coordination polymers, Cr(II)-based molecular coordination cages (also known as metal–organic polyhedra, MOPs) have been gradually developed ever since the pioneering reports by Bloch and co-workers^24^ and Zhou and co-workers^25^ (Figure 1a). The subsequent findings in this area involved preparation of a series of molecular Cr(II)-based coordination cages using a variety of dicarboxylate linkers, which demonstrated their significant structural diversity and promising application potential.^26−28^ For example, similarly to the Cr(II)-MOFs, the Cr(II)-MOPs show excellent O_2_/N_2_ adsorption selectivity. Interestingly, Zhou *et.al.* demonstrated that the gas adsorption properties of Cr(II)-based MOPs changed after exposure to oxygen,^25^ which suggests that harnessing their redox reactivity might be a way to tailor their application properties.

In some cases, MOFs and MOPs are constructed using the same starting materials, which raises the issue related to the synthesis selectivity. For example, depending on the synthetic conditions, copper isophthalates are known to form 3D MOFs,^29^ and 2D-layered MOFs involving both hexagonal (kagomé-type)^30,31^ and square grid frameworks^32^ as well as molecular Cu_24_(m-bdc)_24_ (m-bdc = 1,3-benzenedicarboxylate) cages.^33^ Typically, polymeric 3D and 2D copper isophtalates are prepared by self-assembly at room temperature using various solvent mixtures and/or molecular templates,^34−36^ but some examples of solvothermal synthesis have also been reported.^32^ In turn, the synthesis of molecular Cu_24_(m-bdc)_24_ MOP most often requires increased temperatures (around 80 °C).^33,37,38^ In the case of Cr(II)-based MOPs, the Cr_24_(m-bdc)_24_ cluster can be formed at room temperature by mixing DEF solutions of chromium(II) acetate and isophthalic acid,^25^ but preparation of other Cr(II)-based coordination cages requires variety of different synthetic strategies.^24,26−28^ Notably, all of the previously reported procedures involving Cr(II) salts self-assembled using isophthalates or their derivatives resulted in the formation of cage-type species.^24,25^

As part of our continuing research on the design, synthesis, and functionalization of MOFs^20,39−43^ and following our previous findings on the preparation of Cr(II)-MOFs,^20^ herein, we demonstrate that the selectivity of self-assembly of Cr^2+^ species with isophthalate linkers can be shifted toward formation of the 2D-layered MOF material [Cr(m-bdc)]·H_2_O (**1·H**_**2**_**O**) using the slow diffusion strategy (Figure 1b). Moreover, we investigated the postsynthetic redox reactivity of the developed 2D MOF, which resulted in significant changes in its gas adsorption properties. In particular, we discovered that oxidation of **1** using nitric oxide (NO) or dry oxygen yields porous materials with excellent H_2_/N_2_ gas adsorption selectivity.

## Results and Discussion

The diffusion-controlled synthetic approach to the preparation of MOFs is a very promising strategy, which involves self-assembly at very low concentrations of substrates achieved by slow diffusion of metal ions and linker species. This type of controlled reaction environment provides enough time and energy for the molecules to assemble into high-quality MOF crystals, which are challenging to obtain via alternative methods, as discussed in our previous reports.^20,41^ In particular, a low-temperature diffusion-controlled approach could be a beneficial strategy for controlling the selectivity of self-assembly processes due to limited thermal energy of the reactants. To this end, we decided to apply the diffusion-controlled synthetic approach to the reaction system involving Cr(II) ions and isophtalate linkers (m-bdc), which could lead to the formation of MOFs or MOPs, as inferred by comparison to Cu(II)-based analogues.

**Figure 2:**
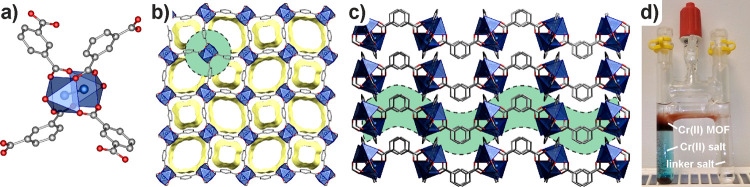
(a) Paddlewheel [Cr_2_(OOCR)_4_] secondary building unit in **1·H**_**2**_**O**; (b) 2D MOF layer in **1·H**_**2**_**O** showing two different-sized pores (in yellow); green area shows the paddlewheel unit; (c) AA-type stacking of the 2D MOF layers in the crystal lattice of **1·H**_**2**_**O**; green area shows the individual 2D layer. Cr = blue, O = red, and C = gray, H atoms have been omitted for clarity; (d) custom-made glass reactor for the diffusion-controlled synthesis of Cr(II) MOFs.

The reaction between CrSO_4_ and Na_2_(m-bdc) in water conducted at 50 °C in diffusion-controlled conditions (using a custom-made glass reaction cell shown in Figure 2d) resulted in the formation of red crystalline product: [Cr(m-bdc)]·H_2_O (**1·H**_**2**_**O**). Single-crystal X-ray diffraction experiment revealed that **1·H**_**2**_**O** crystallizes in the *P*-42_1_m space group and forms a layered 2D MOF structure (Figure 2 a–c, Tables S1 and S2). The individual MOF layers involve Cr_2_(O_2_CR)_4_ paddlewheel-type units with water molecules coordinated to each of the metal centers (Figure 2a). The Cr–Cr distance of 2.2960(18) Å in **1·H**_**2**_**O** is close to that observed for other chromium(II) carboxylate paddlewheels with axial water ligands reported previously showing Cr–Cr distances in the range of 2.33–2.36 Å.^20,44^ The chromium paddlewheel units are interconnected by isophthalate linkers forming grid-type assemblies, which are AA-type stacked with a layer-to-layer distance of 6.702 Å, slightly shorter in comparison to that observed for the isostructural copper-based MOFs showing the layer-to-layer distance in the range of 6.74–6.79 Å.^32,45^ Due to the orientation of the linker backbone, the 2D layers of Cr(m-bdc)]·H_2_O involve two types of openings, which extend into 1D channels (pore limiting diameters: 5.59 and 3.84 Å; maximum pore diameters: 6.74 and 5.09 Å, calculated using the Poreblazer v4.0 software^46^) perpendicular to the coordination polymer layers (Figure 2b). The phase purity of the prepared material was confirmed by PXRD (Figure S2). Notably, the increased reaction temperature (50 °C) was necessary for the efficient preparation of **1·H**_**2**_**O** since analogous reaction conducted at room temperature resulted in the formation of a mixture of products with overall lower crystallinity, as evidenced by the PXRD study (Figure S3).

**Figure 3:**
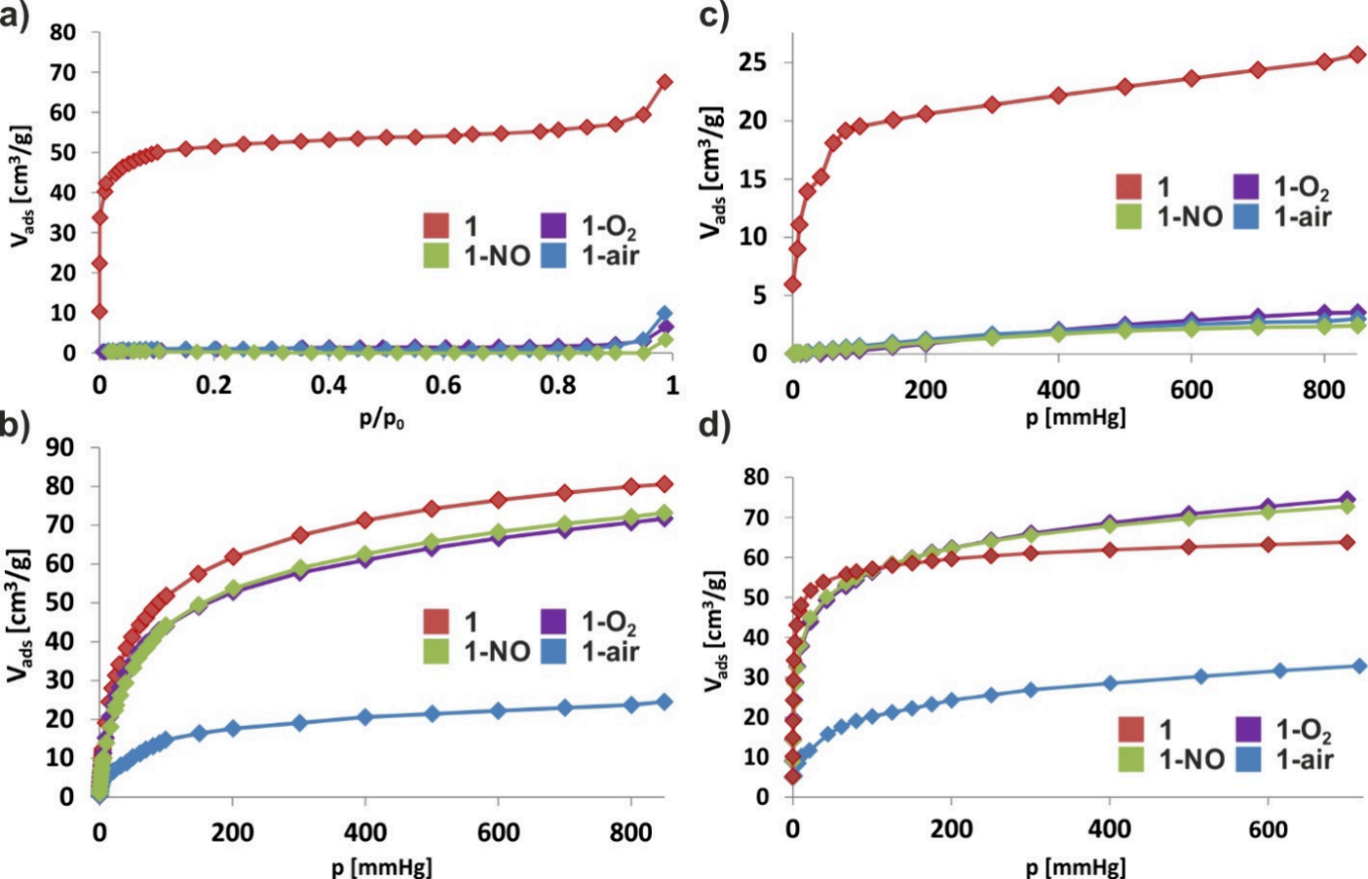
Gas adsorption study of examined materials **1** (red), **1-NO** (green), **1-O**_**2**_ (purple), and **1-air** (green): (a) N_2_ adsorption isotherms (at 77 K), (b) H_2_ adsorption isotherms (at 77 K), (c) O_2_ adsorption isotherms (at 273 K), and (d) CO_2_ adsorption isotherms (at 195 K).

The presence of 1D channels in the crystal structure of **1·H**_**2**_**O** prompted us to conduct gas adsorption experiments in order to evaluate the application potential of the material. The initial activation tests showed that relatively high temperature (200 °C) and vacuum treatment were required to remove solvent molecules from the pores, which yielded activated material **1**. The nitrogen adsorption experiment of **1**, conducted at 77 K, revealed a type I isotherm typical for microporous materials with a maximum uptake of 67 cm^3^/g of STP and a BET surface area of 197 m^2^/g (Figure 3a, red curve). The hydrogen adsorption experiments conducted at 77 and 87 K (Figures 3b and S7) confirmed the microporosity of **1** revealing the maximum uptakes of 80.5 cm^3^/g and 68.6 m^3^/g, respectively, and the zero-coverage H_2_ heat of adsorption of 7.4 kJ/mol (Figure S10), which is slightly higher in comparison to the value 6.2 kJ/mol reported for Cr_3_(BTC)_2_ MOF.^20^ Additionally, CO_2_ adsorption experiments were conducted at −78, 0, and 20 °C, which showed 63.8, 40.4, and 32.2 cm^3^/g maximum uptakes, respectively (Figures 3d, S5 and S6).

**Figure 4:**
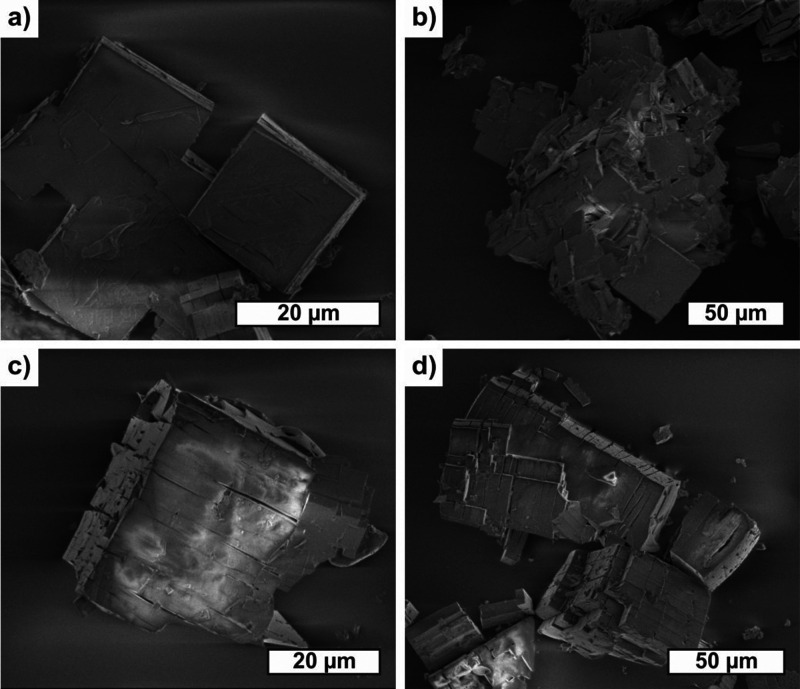
SEM images of **1·H**_**2**_**O** (a,b) and **1-NO** (c,d).

With regard to the literature data,^16,20,25^ the high redox reactivity of the chromium centers in **1** was expected, which could provide an opportunity to perform redox-based postsynthetic modification and potentially tailor the properties of the developed material. To this end, we selected three sources of oxidative species, nitric oxide (NO), dry oxygen, and air, and used them to oxidize the developed Cr(II)-based MOF, yielding three modified materials, **1-NO**, **1-O**_**2**_ and **1-air**, which were further investigated using PXRD, spectral analyses, and gas adsorption studies. Freshly prepared samples of **1·H**_**2**_**O** were exposed to NO, O_2_, or air for 12 h at RT, which resulted in a gradual color change (Figure S1) from red to brown (sample **1-NO**) or green (samples **1-O**_**2**_ and **1-air**). The exposure of **1·H**_**2**_**O** to NO followed by evacuation resulted in the sample weight increase by 11.2%, which is close to the theoretical value of 12.8% representing stochiometric 1:1 binding of the NO molecule to each Cr(II) center. The **1-NO** sample composition was confirmed using elemental analysis, revealing the averaged formula as [**1**· 0.82 H_2_O · 0.91 NO]. Furthermore, the exposure of **1·H**_**2**_**O** to dry oxygen or atmospheric air followed by evacuation resulted in a sample weight increase of 2.5% and 5.4%, respectively, while the theoretical values calculated for [**1**·H_2_O·O_2_] and [**1**·O_2_] were 13.7% and 6.0%, respectively. Based on the elemental analysis, the averaged formulas of materials **1-O**_**2**_ and **1-air** were calculated as [**1**· 0.45 H_2_O · 0.52 O_2_] and [**1**· 0.92 H_2_O · 0.43 O_2_], respectively. Materials **1·H**_**2**_**O**, **1-NO**, **1-O**_**2**_, and **1-air** were studied using solid-state diffuse reflectance UV–vis spectroscopy, which suggested that the color changes observed upon exposure of **1·H**_**2**_**O** to NO, O_2_, or air were related to redox reactions of the chromium centers rather than formation of charge transfer complexes due to the absence of characteristic charge-transfer bands (Figure S16). The PXRD analysis of the oxidized samples showed that the crystallinity of the studied material dropped significantly (Figure S4), *i.e.*, sample **1-NO** was essentially amorphous and samples **1-O**_**2**_ and **1-air** retained the original long-range arrangement, but the observed reflections were broader and less intense in comparison to the crude **1·H**_**2**_**O**. This observation clearly indicates that oxidation of Cr(II)-MOF results in the introduction of major structural defects. Moreover, scanning electron microscopy (SEM) images of materials **1** and **1-NO** revealed that in spite of amorphization, the grain size and shape before and after oxidation were very similar (regularly shaped grains ca. 10–50 μm in size). However, significant structural damage (fissures and cracks) was introduced to the material grains upon oxidation with NO, as demonstrated in the SEM pictures (Figures 4, S13, and S14).

Gas adsorption experiments of the oxidized materials demonstrated promising improvements in the H_2_/N_2_ adsorption selectivity in comparison to that of **1**. The maximum H_2_ adsorption uptakes of samples **1-NO**, **1-O**_**2**_, and **1-air** were 73.2, 71.7, and 24.5 cm^3^/g (Figure 3b, green, purple, and blue curves), which correspond to ca. 91, 89, and 30% of the original capacity of **1**, respectively, but their ability to adsorb N_2_ at 77 K was essentially not observed (Figure 3a, green, purple, and blue curves). While in the case of material **1-air,** the observed decrease in the gas adsorption properties can be attributed to the sample decomposition, the properties of the samples **1-NO** and **1-O**_**2**_ indicate that in spite of the amorphization, the resulting material acts as a molecular sieve for the H_2_/N_2_ mixture. Further H_2_ adsorption experiments conducted at 87 K revealed maximum uptakes of 68.6, 56.1, and 58.8 cm^3^/g for **1**, **1-NO,** and **1-O**_**2**_, respectively (Figures S7 – S9) and zero coverage isosteric heats of adsorption (Qst) for H_2_ of 7.4, 7.2, and 7.6 kJ mol, respectively (Figure S10). Moreover, cyclic experiments of H_2_ adsorption by **1-NO** revealed excellent repeatability of the observed uptakes (Figure S11). Notably, despite the significant industrial interest in H_2_/N_2_ separation,^7^ only a handful of MOFs capable of molecular sieving of H_2_/N_2_ mixture have been reported till date, including 2D^47,48^ and 3D^49−51^ systems. In order to compare the performance of the **1-NO** material to the previously known MOF-based H_2_/N_2_ molecular sieves (materials showing significant H_2_ adsorption and negligible N_2_ adsorption), we have selected the maximum H_2_ loading capacity at 77 K as the benchmark parameter. As demonstrated in Table S3, **1-NO** and **1-O**_**2**_ exhibit properties well within the range of the best currently known MOF-based H_2_/N_2_ molecular sieves.

Further experiments involving CO_2_ adsorption revealed that the performance of **1-NO**, **1-O**_**2**_, and **1-air** dropped with respect to **1** in experiments conducted at 0 and 20 °C. The maximum CO_2_ uptakes of **1-NO, 1-O**_**2**_, and **1-air** were 32.8, 34.6, and 24.4 cm^3^/g at 0 °C and 24.0, 25.1, and 17.9 cm^3^/g at 20 °C, respectively, which are in the range of 56–86% of the reference values observed in **1** (Figures S5 and S6). However, CO_2_ adsorption experiments conducted at −78 °C showed that both **1-NO** and **1-O**_**2**_ overperformed the original material **1** exhibiting maximum CO_2_ capacities of 72.7 and 74.6 cm^3^/g (Figure 3d). Finally, since the Cr(II)-based porous materials are known to have promising O_2_ adsorption properties,^16,25^ we measured the O_2_ adsorption isotherms at 0 °C for **1**, **1-NO**, **1-O**_**2**_, and **1-air** materials. As a result, it was found that material **1** showed promising O_2_ adsorption capacity of 25.7 cm^3^/g, but the adsorption process was not reversible, as evidenced by the repeated experiment reaching the capacity of only 3.7 cm^3^/g (Figure S12), which is similar to the results reported by Zhou for the Cr(II)-based MOP.^25^ Moreover, the O_2_ adsorption capacities of the preoxidized samples **1-NO**, **1-O**_**2**_, and **1-air** showed maximum capacities in the range 3–4 cm^3^/g (Figure 3c).

Due to the low crystallinity of **1-NO**, **1-O**_**2**_, and **1-air** materials, detailed structural investigations aimed at deeper understanding of the observed adsorption phenomena were significantly hampered. Nevertheless, in order to probe the structures of **1-NO**, **1-O**_**2**_, and **1-air**, FTIR spectroscopy was employed. Comparison of the FTIR spectra of **1** before and after the oxidation with NO revealed the emergence of new signals at 1858 and 1714 cm^–1^ (Figure S15), which could be attributed to the stretching vibrations of nitrosyl groups bonded to the Cr(III) centers.^52−55^ These observations indicate that upon exposure to gaseous NO, the Cr^(II)^ centers in **1** were oxidized forming the Cr^(III)^-NO species. Furthermore, the FTIR spectra of samples **1-O**_**2**_ and **1-air** appear very similar, but their complexity hindered the unequivocal identification of the specific chemical structure of the chromium–oxygen species present in these samples.

## Conclusions

In summary, we would like to emphasize that the redox-active Cr(II)-based MOFs show great potential for both basic and applied research, but their investigation poses significant experimental challenges, as reflected by only a handful of reports following the pioneering work by Long and co-workers.^16^ While previously reported attempts at self-assembly of Cr^2+^ cations and isophthalate linkers led to the formation of molecular MOP cages,^25^ our study shows that using the diffusion-controlled approach allows for directing the course of this process toward a novel 2D MOF product. Moreover, we developed a promising strategy for tailoring the gas adsorption properties of the Cr(II)-based MOF by capitalizing on its redox reactivity, which resulted in the preparation of a material with excellent H_2_/N_2_ adsorption selectivity. Thus, the presented results provide valuable insights into the relatively unexplored field of Cr(II)-MOFs. They also demonstrate a promising strategy of MOFs' postsynthetic modification utilizing their redox reactivity, which could inspire more investigations in this promising research area. Further studies involving other redox-reactive MOFs are currently underway.

## Experimental Section

### Materials and Methods

All reactions and manipulations were carried out under an inert atmosphere of dry nitrogen using standard Schlenk and glovebox techniques. Water was prepared by repeated sonication, vacuum treatment, and N_2_ bubbling in order to fully remove dissolved O_2_. THF was distilled off with sodium benzophenone immediately prior to use. Sodium hydroxide and 1,3-benzenedicarboxylic acid were purchased from Aldrich and used as received. Sodium 1,3-benzenedicarboxylate was prepared by mixing appropriate amounts of NaOH and carboxylic acids in water. Chromium(II) sulfate was prepared according to a literature procedure.^56^

### Synthesis of **1·H_2_O**

Solutions of chromium(II) sulfate pentahydrate (690 mg, 2.9 mmol) in 2 mL of water and sodium 1,3-benzenedicarboxylate [freshly prepared using 481 mg (2,9 mmol) of 1,3-benzenedicarbocylic acid and 232 mg (5.8 mmol) of NaOH] in 2 mL of water were prepared. The solutions were placed in a special glass reactor that prevented mixing of the substrates (Figure 1d). Next, the reactor was gently filled with water, creating a diffusion path between substrates. The diffusion-controlled process was conducted for 4 weeks at 50 °C, which resulted in the formation of red single crystals of **1·H**_**2**_**O**. The product was collected, washed three times with water and THF, and dried in vacuo. PXRD analysis confirmed the phase purity of the product. Yield: 497 mg (73%); elemental analysis of **1·H**_**2**_**O**: C, H (%) calcd for Cr_1_C_8_H_6_O_5_: C 41.04, H 2.58; found C 41.23, H 2.62.

### Synthesis of **1-NO**

Sample of **1·H**_**2**_**O** (100 mg) was exposed to gaseous NO at atmospheric pressure and room temperature for 12 h, followed by evacuation. As a result, 111.2 mg of brown powder was obtained. Elemental analysis of **1-NO** C, H (%) calcd for [CrC_8_H_4_O_4_· 0.82 H_2_O · 0.91 NO]: C 37.22, N 4.94, H 2.20; found C 37.21, N 4.95, H 2.21.

### Synthesis of **1-O_2_**

Sample of **1·H**_**2**_**O** (100 mg) was exposed to dry oxygen at atmospheric pressure and room temperature for 12 h, followed by evacuation. As a result, 102.5 mg of green powder was obtained. Elemental analysis of **1-O**_**2**_ C, H (%) calcd for [CrC_8_H_4_O_4_· 0.45 H_2_O · 0.52 O_2_]: C 39.89, H 2.05; found C 39.87, H 2.06.

### Synthesis of **1-air**

Sample of **1·H**_**2**_**O** (100 mg) was exposed to atmospheric air at atmospheric pressure and room temperature for 12 h, followed by evacuation. As a result, 105.4 mg of green powder was obtained. Elemental analysis of **1-air** C, H (%) calcd for [CrC_8_H_4_O_4_· 0.92 H_2_O · 0.43 O_2_]: C 38.99, H 2.39; found C 38.98, H 2.41.

### X-ray Crystallography

The crystals were selected under Paratone-N oil, mounted on nylon loops, and positioned in cold stream on the diffractometer. The X-ray data for **1·H**_**2**_**O** were collected at 100(2) K on a SuperNova Agilent diffractometer using Cu*Kα* radiation (λ = 1.54184 Å). The data were processed with *CrysAlisPro.*(57) Structures were solved by direct methods and refined using *SHELXL-2016/4.*(58) All non-hydrogen atoms were refined with anisotropic displacement parameters. Hydrogen atoms were added to the structure model at geometrically idealized coordinates and refined as riding atoms. In **1·H**_**2**_**O,** no satisfactory structural models for highly disordered solvent molecules could be assigned, and therefore, Solvent Masking in OLEX2 was used to remove the electron densities of these disordered species.^59^

### PXRD Analysis

PXRD data were collected using an Empyrean diffractometer (PANalytical) employed with Ni-filtered Cu Kα radiation (40 kV, 40 mA) using Bragg–Brentano geometry. In order to achieve air-free conditions for measurements of sensitive samples, Anton-Paar PEEK polymer domed sample holders were used, which were loaded in an Ar-filled glovebox. Samples were mounted on zero-background silicon sample holders prior to measurements.

### Gas Adsorption Analysis

Volumetric gas sorption studies were undertaken using a Micromeritics Instrument Corporation (Norcross, Georgia, USA) ASAP 2020 system. Approximately, 100 mg of the corresponding solid product was transferred to a preweighed sample tube and evacuated under vacuum at 200 °C on the gas adsorption apparatus until the outgas rate was <5 μmHg. All gases used were of 99.999% purity. Helium was used for the freespace determination after sorption analysis. Adsorption isotherms were measured at 77 K in a liquid nitrogen bath. Temperatures of 273 and 293 K for CO_2_ isotherms were maintained with a thermostated external ethylene glycol bath.

### SEM Imaging

SEM imaging was performed on a FEI Nova NanoSEM 450 microscope with a field-emission gun utilizing ETD and TLD detectors. High-quality imaging was performed with electron beam energy varying from 1 to 2 kV.

### Infrared Spectroscopy

Infrared spectra were collected using a Bruker Tensor apparatus equipped with an ATR accessory. Samples were prepared by mixing ca. 1 mg of MOF with nujol in order to prevent air exposure. Resulting suspensions were transferred to the ATR sample stage and scanned 16 times in the wavenumber range from 400 to 4500 cm^–1^.

### UV–Vis Spectroscopy

UV–vis diffuse reflectance spectroscopy was performed at room temperature using a UV-2600 Shimadzu spectrophotometer in the spectral range of 187–1400 nm. For the nonabsorbing reflecting material, BaSO_4_ was used as a reference. The samples were prepared in an Ar-filled glovebox using a sealable polymer sample holder with a quartz window in order to prevent air exposure.

## Supporting Information Available

Additional pictures, graphs, and tables concerning synthesis, SCXRD, PXRD, gas adsorption measurements, SEM, and FTIR spectroscopy (PDF)
Single-crystal data (CIF)

## Supplementary Material

am4c06228_si_001.pdf

am4c06228_si_002.cif
