# Supplementary material for: Chromium(II)-isophthalate 2D MOF with Redox-Tailorable Gas Adsorption Selectivity
Source: ACS Appl Mater Interfaces. 2024 Aug 19;16(34):45100–6. doi: 10.1021/acsami.4c06228 (PMC11367576; doi:10.1021/acsami.4c06228)
Supplement: Supplementary file 1 — am4c06228_si_001.pdf [file am4c06228_si_001.pdf]

Supporting information

Chromium(II)-isophthalate 2D MOF with redox-tailorable gas adsorption selectivity

Michał K. Leszczyński,*[,a,b] Katarzyna Niepiekło,[a] Michał Terlecki,[a] Iwona Justyniak,[b] and Janusz Lewiński*[,a,b]

[a] Faculty of Chemistry, Warsaw University of Technology, Noakowskiego 3, 00-664 Warsaw (Poland),

[b] Institute of Physical Chemistry, Polish Academy of Sciences, Kasprzaka 44/52, 01-224 Warsaw (Poland)

Supplementary Information (15 pages)

Table of contents:

1. Synthesis – Figure S1 2
2. Single crystal X-Ray crystallography – Tables S1-S2 3
3. PXRD analysis – Figures S2 – S4 4
4. Gas adsorption analysis – Figures S5 – S12, Table S3 6
5. SEM imaging – Figures S13 – S14 11
6. Infrared spectroscopy – Figure S15 13
7. UV-Vis spectroscopy – Figure S16 14
8. References 15

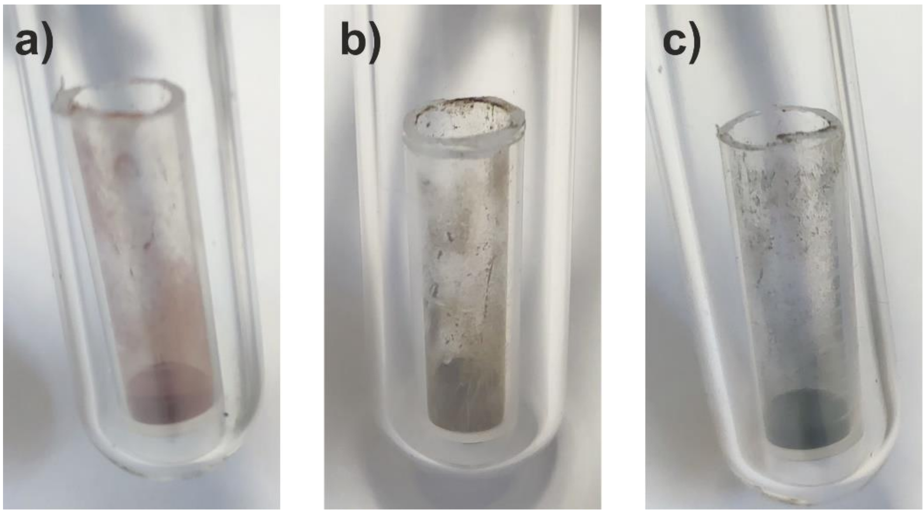

Figure S1. Pictures of **1·H_2O** (a), **1-NO** (b) and **1-O_2** (c).

Table S1. Crystal data and structure refinement for **$1\cdot H_2O$**.

Identification code	**$1\cdot H_2O$ (CCDC – 2291621)**	
Empirical formula	C8 H6 Cr O5	
Formula weight	234.13	
Temperature	100(2) K	
Wavelength	1.54184 Å	
Crystal system	Tetragonal	
Space group	*P* -4 2_1 m	
Unit cell dimensions	a = 19.2322(4) Å	α = 90°.
	b = 19.2322(4) Å	β = 90°.
	c = 6.7019(2) Å	γ = 90°.
Volume	2478.88(13) $Å^3$	
Z	8	
Density (calculated)	1.255 Mg/m^3	
F(000)	944	
Crystal size	0.14 x 0.09 x 0.0 mm^3	
Theta range for data collection	4.598 to 69.994°.	
Index ranges	-23<=h<=22, -14<=k<=23, -8<=l<=8	
Reflections collected	13029	
Independent reflections	2438 [R(int) = 0.141]	
Completeness to theta = 25.242°	99.2 %	
Max. and min. transmission	0.684 and 0.462	
Refinement method	Full-matrix least-squares on F^2	
Data / restraints / parameters	2438 / 0 / 136	
Goodness-of-fit on F^2	1.078	
Final R indices [I>2sigma(I)]	R1 = 0.0651, wR2 = 0.1775	
R indices (all data)	R1 = 0.0674, wR2 = 0.1800	
Extinction coefficient	n/a	
Largest diff. peak and hole	0.865 and -0.467 e. $Å^{-3}$	

Table S2. Selected atomic distances [Å] in **1**.

Cr1-Cr1	2.2960(18)	Cr1-O1	2.0024(58)
Cr1-O1’	2.0030(44)	Cr1-O4	2.0081(57)
Cr1-O4’	2.0087(57)	Cr1-O5	2.2653(70)
Cr2-O2	2.0174(44)	Cr2-O2’	2.0180(58)
Cr2-O3	1.9933(57)	Cr2-O3’	1.9939(42)
Cr2-O6	2.2468(85)		

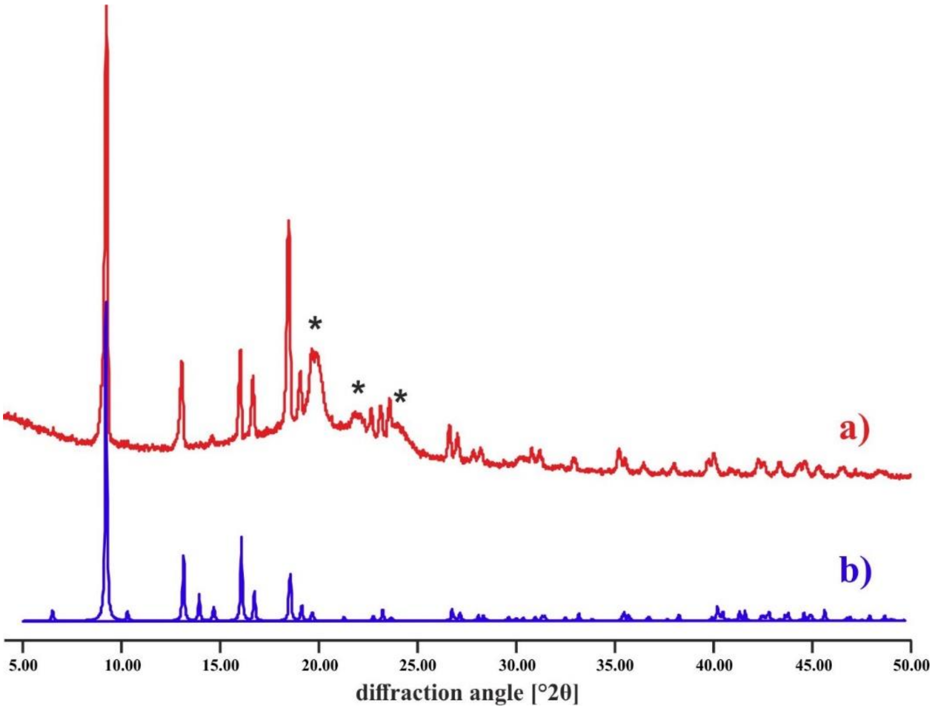

Figure S2. a) Experimental PXRD diffractogram of **1·H_2O** prepared in accordance with the procedure reported in this study, b) simulated PXRD diffractogram if **1·H_2O** (with preferred orientation of crystals). *artifacts originating from the PEEK polymer dome used to achieve air-free conditions during the measurement.

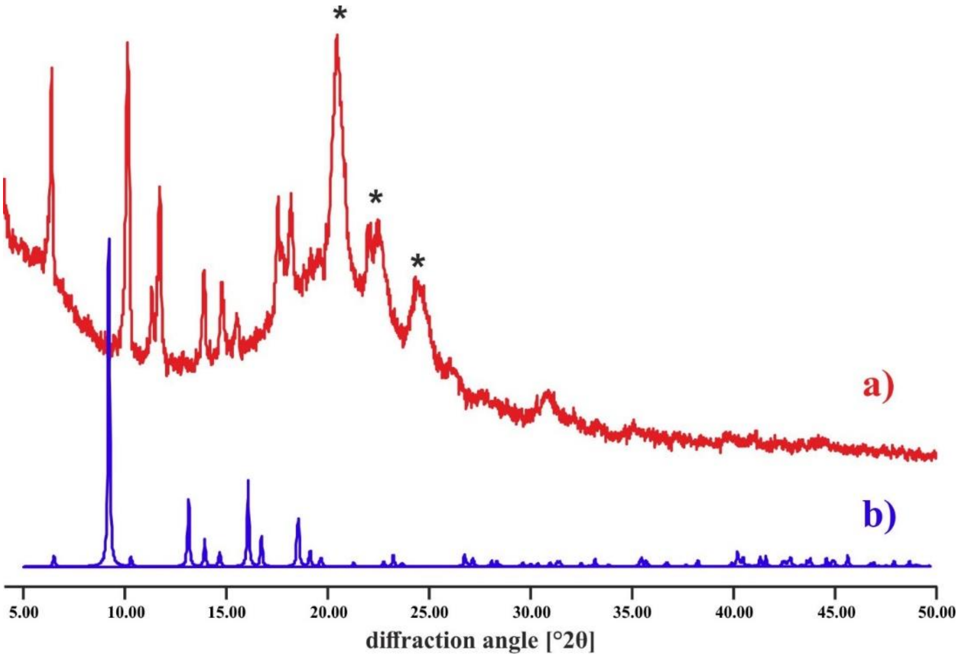

Figure S3. a) Experimental PXRD diffractogram of material prepared following the reported procedure for synthesis of **1·H_2O**, but using temperature 25 °C instead of 50 °C, b) simulated PXRD diffractogram if **1·H_2O** (with preferred orientation of crystals). *artifacts originating from the PEEK polymer dome used to achieve air-free conditions during the measurement.

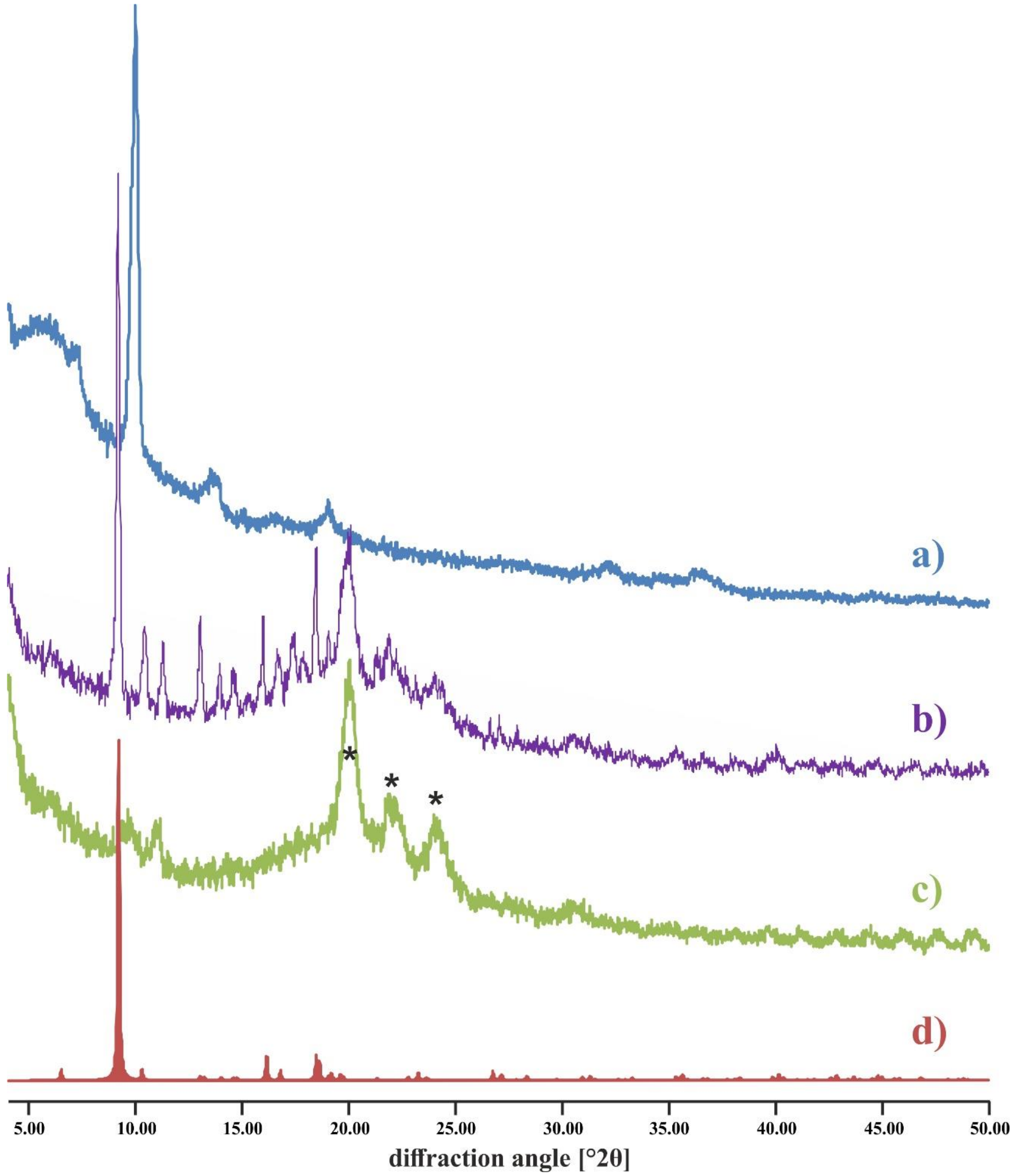

Figure S4. PXRD diffractograms of **1**·**H_2O** oxidation products: **1-air** (a), **1-O_2** (b) and **1-NO** (c) as well as simulated PXRD diffractogram based on the single crystal data of **1**·**H_2O** (d). *****artifacts originating from the PEEK polymer dome used to achieve air-free conditions during the measurement.

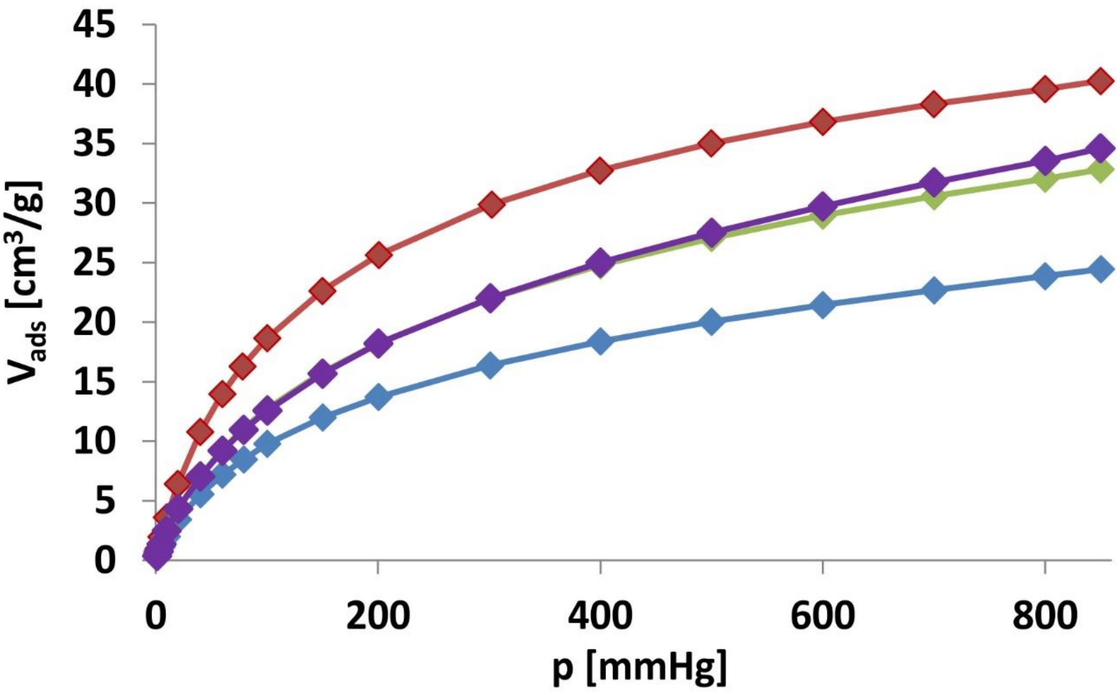

Figure S5. CO_2 adsorption isotherms collected at 0 °C for samples: **1** (red curve), **1-NO** (green curve), **1-O$_2$** (purple curve) and **1-air** (blue curve).

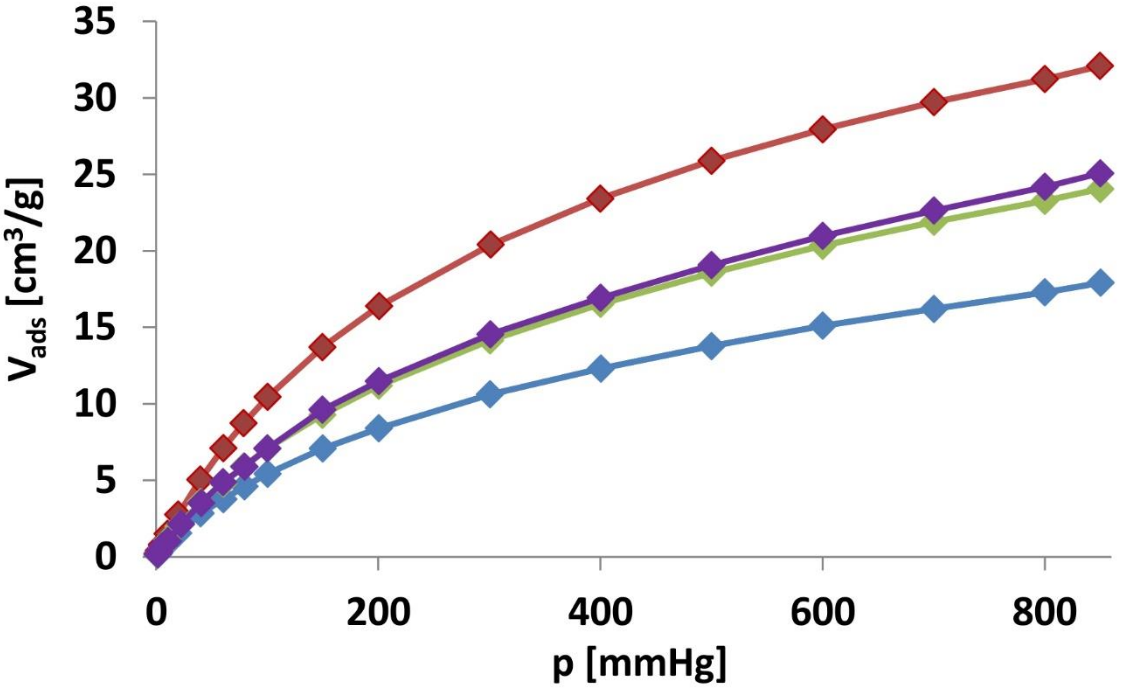

Figure S6. CO_2 adsorption isotherms collected at 20 °C for samples: **1** (red curve), **1-NO** (green curve), **1-O$_2$** (purple curve) and **1-air** (blue curve).

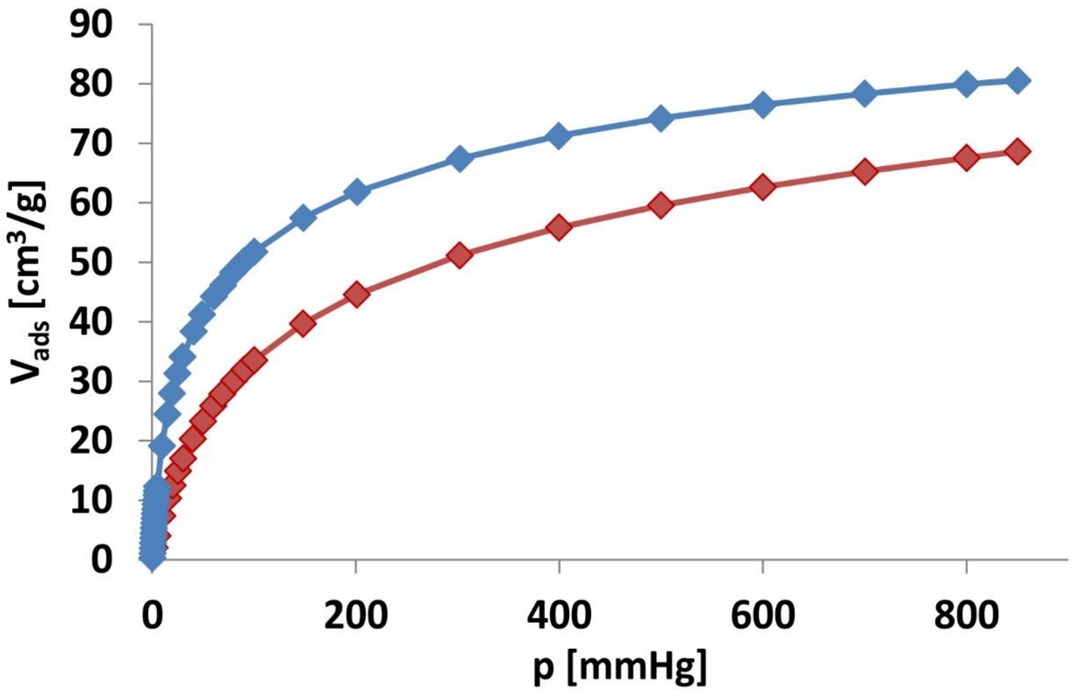

Figure S7. H_2 adsorption isotherms of **1** collected at 77 K (blue curve) and 87 K (red curve).

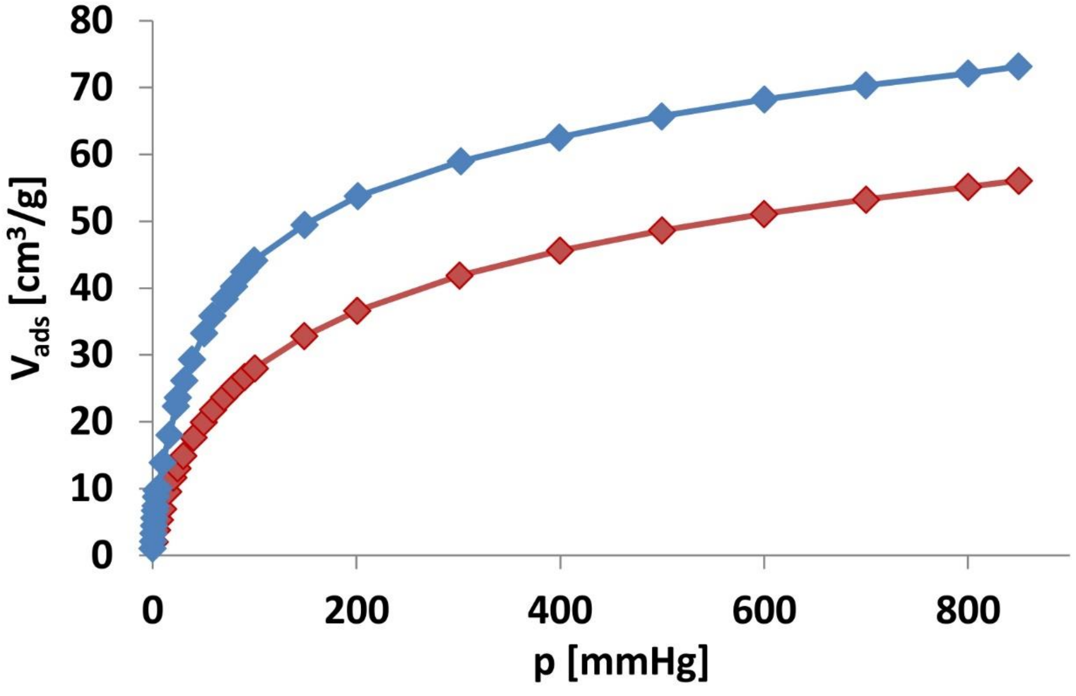

Figure S8. H_2 adsorption isotherms of **1-NO** collected at 77 K (blue curve) and 87 K (red curve).

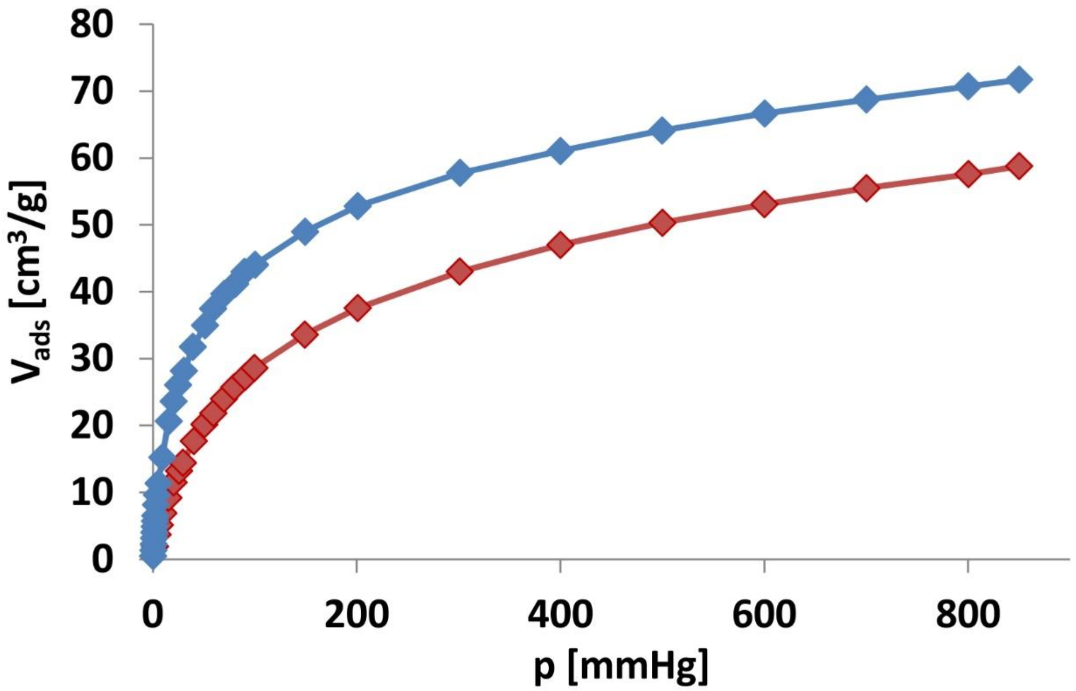

Figure S9. H_2 adsorption isotherms of **1-O₂** collected at 77 K (blue curve) and 87 K (red curve).

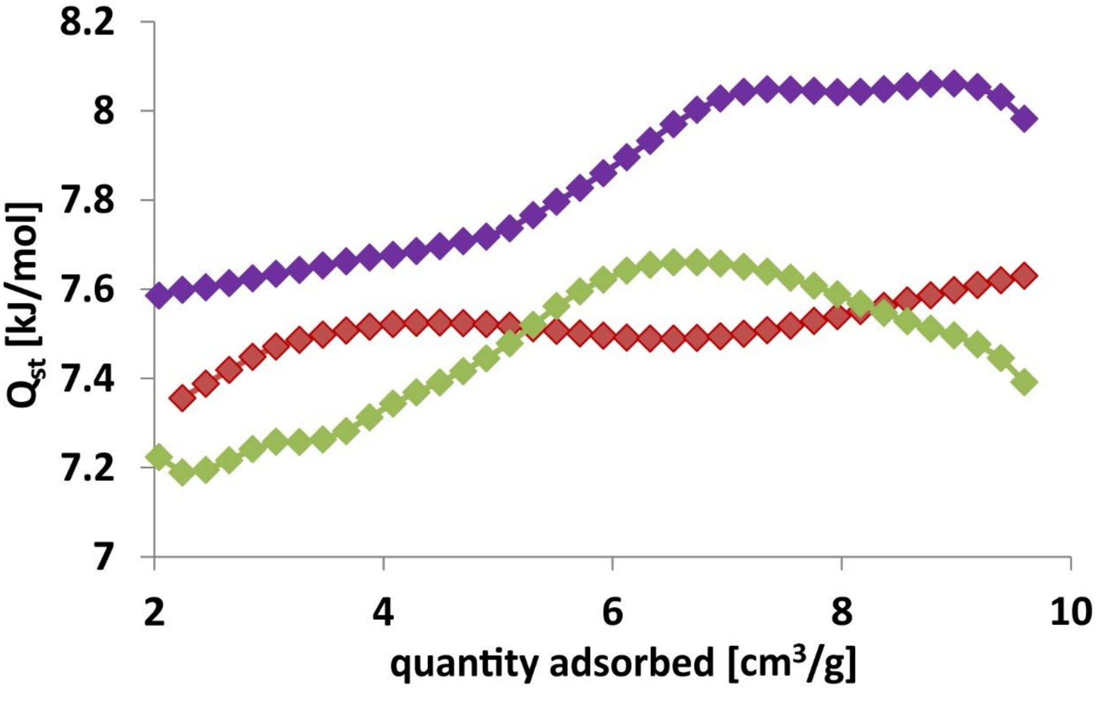

Figure S10. Isosteric heat of absorption of H_2 in **1** (red curve), **1-NO** (green curve) and **1-O_2** (purple curve).

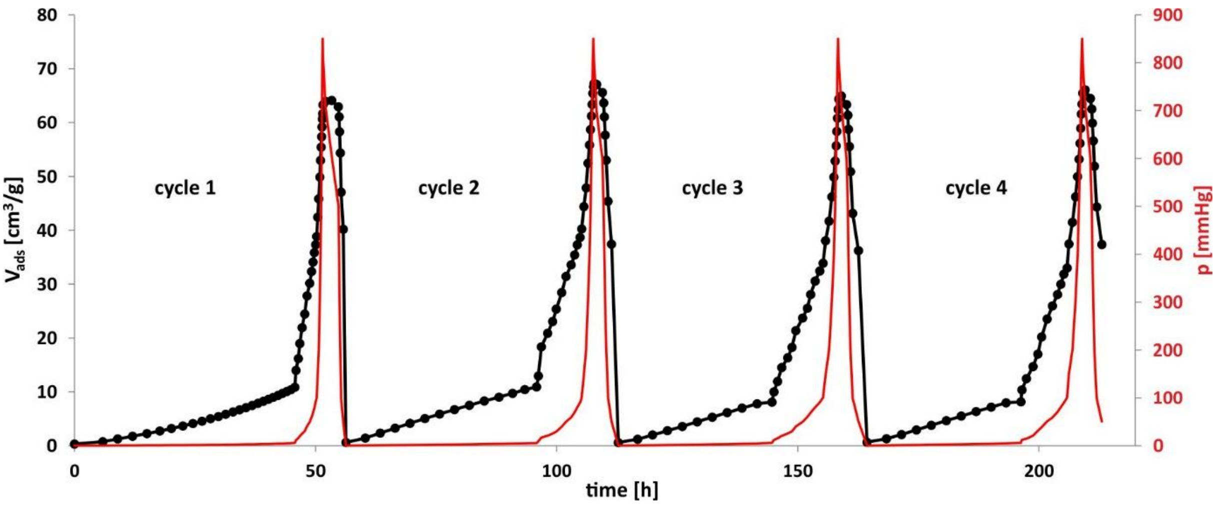

Figure S11. Cycles of repeated H_2 adsorption experiments of **1-NO**, collected at 77 K. Black curve depicts amount of adsorbed gas and red curve depicts the current pressure.

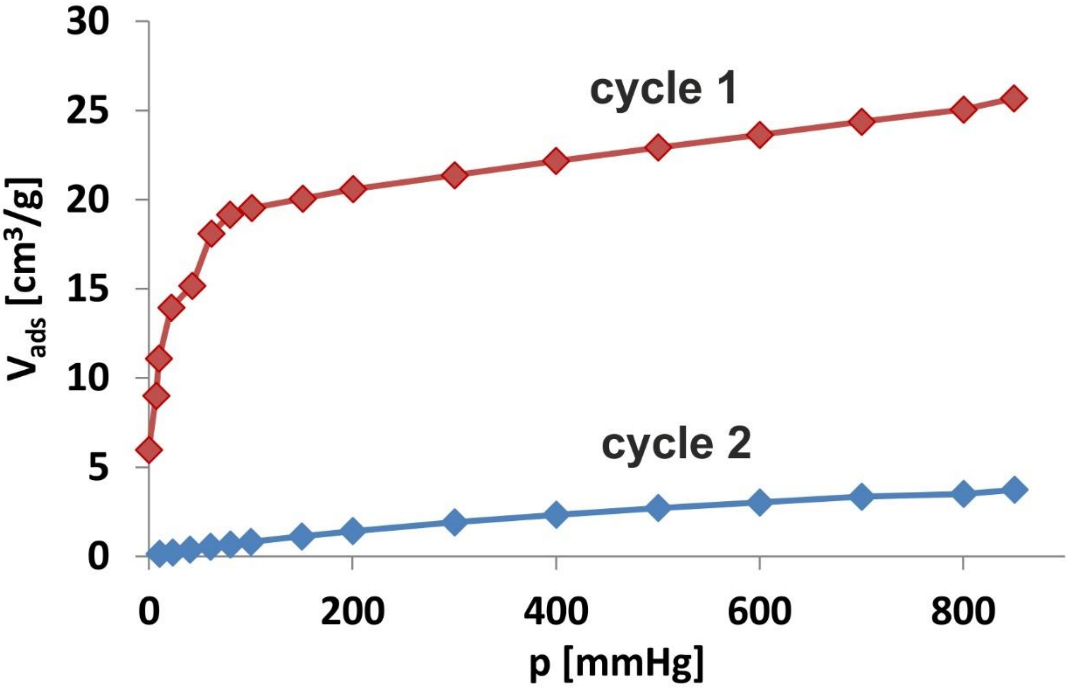

Figure S12. Repeated O_2 adsorption isotherms of **1** collected at 273 K. Initial isotherm = red, repeated isotherm = blue.

Table S3. The top performing known MOF-based H_2/N_2 molecular sieves, based on their maximum H_2 uptake at 77 K.

Material	Maximum H_2 uptake at 77 K [cm^3/g][a]	Reference
**Fe[$Py_2(F_3$-ph$)_2$Por]·Cl·2DMF**	138	1
**Co[$Py_2(F_3$-ph$)_2$Por]·3DMF**	104	1
**Ni[$Py_2(F_3$-ph$)_2$Por]·3DMF**	90	1
**PESD-1**	67	2
**PESD-2**	79	2
**PESD-3**	74	2
**$Mg_3(NDC)_3$**	51	3
**PCN-39(ac)**	45	4
**bnn-1-Ca-H_2O**	56	5
**bnn-1-Ca**	62	5
**1-NO**	73	*this work*
**1-O_2**	72	*this work*

[a]Reported values were acquired by digitisation of the isotherm data.

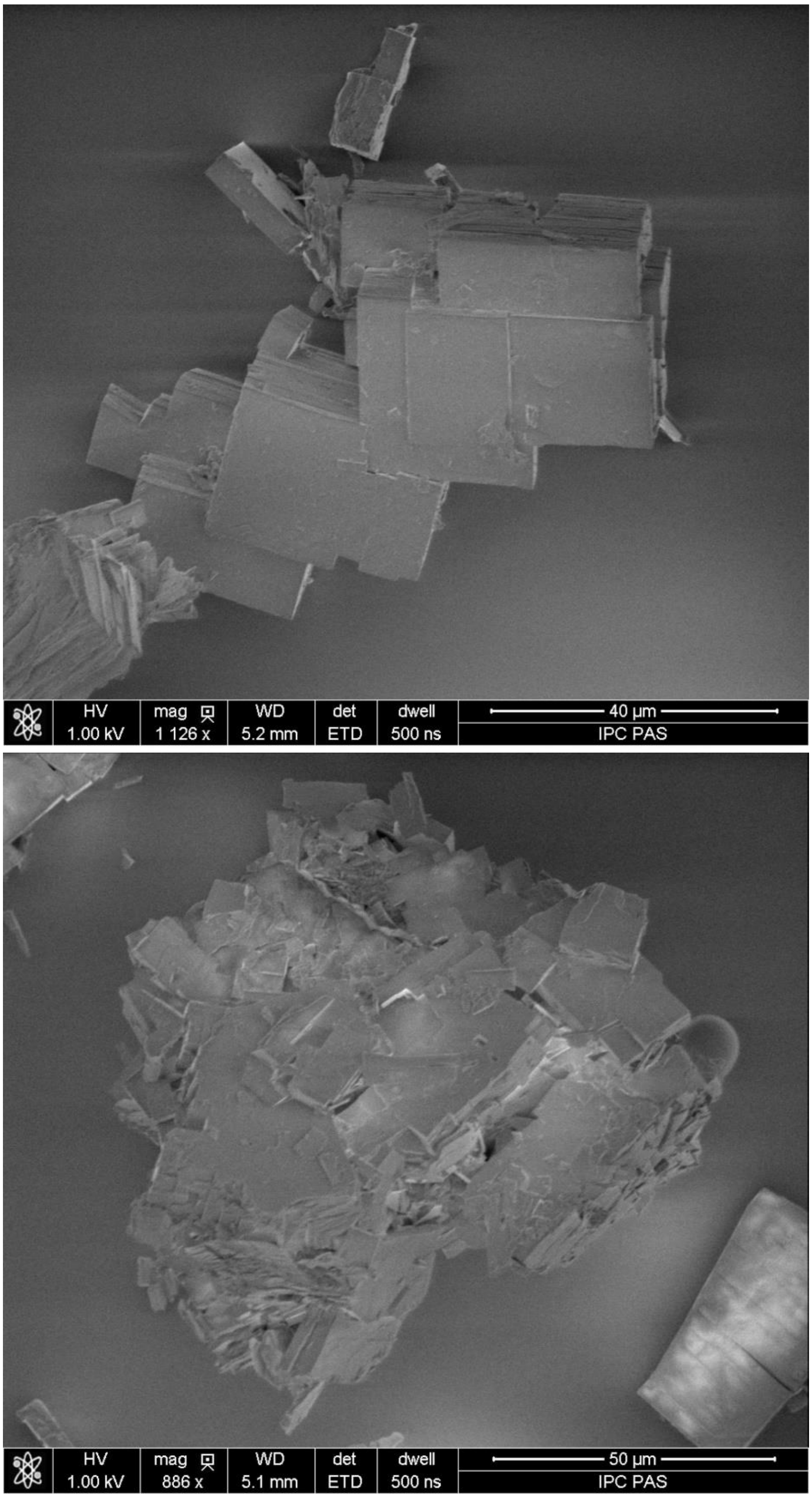

Figure S13. SEM micrographs of **1·H_2O**.

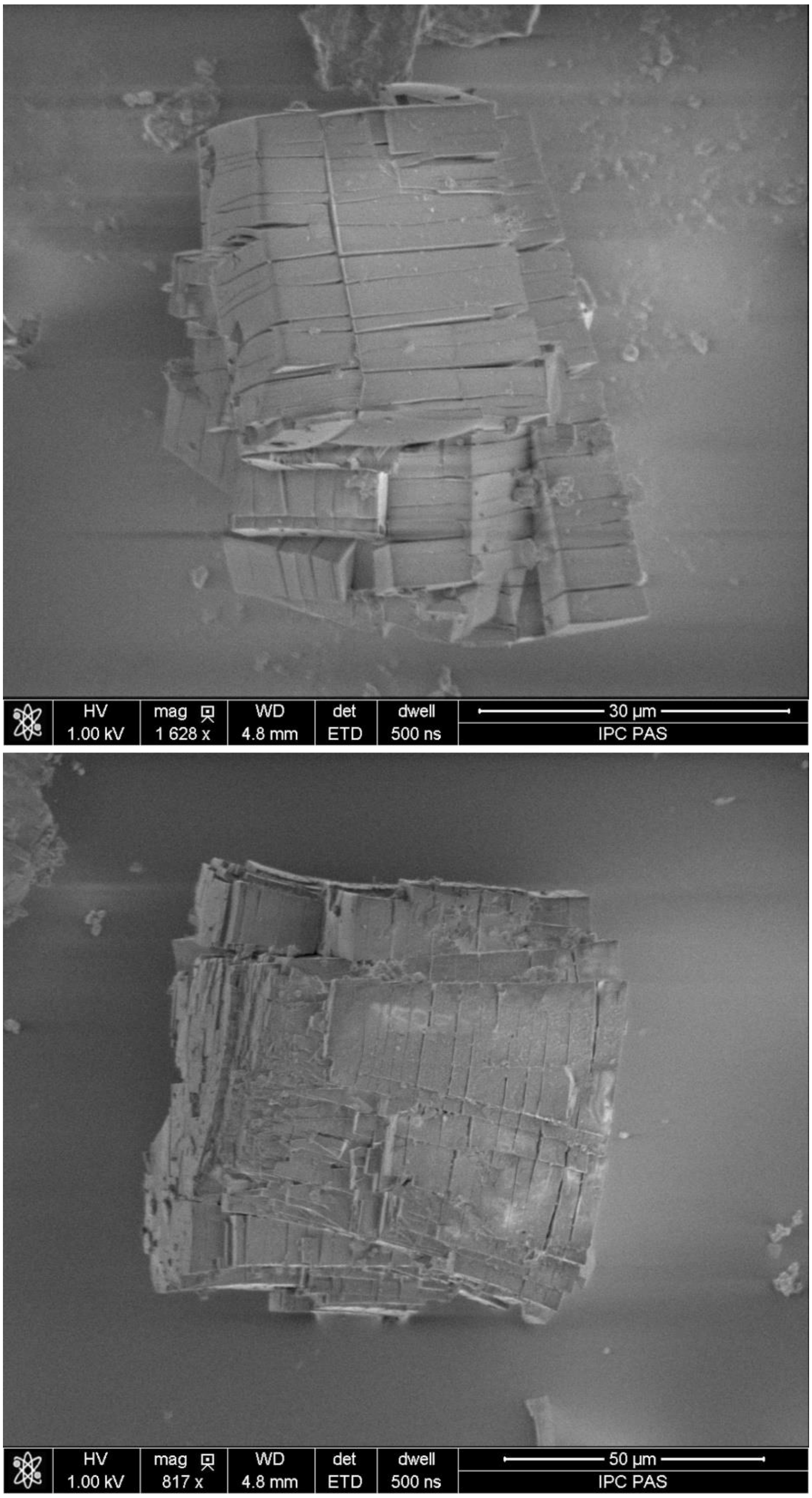

Figure S14. SEM micrographs of **1-NO**.

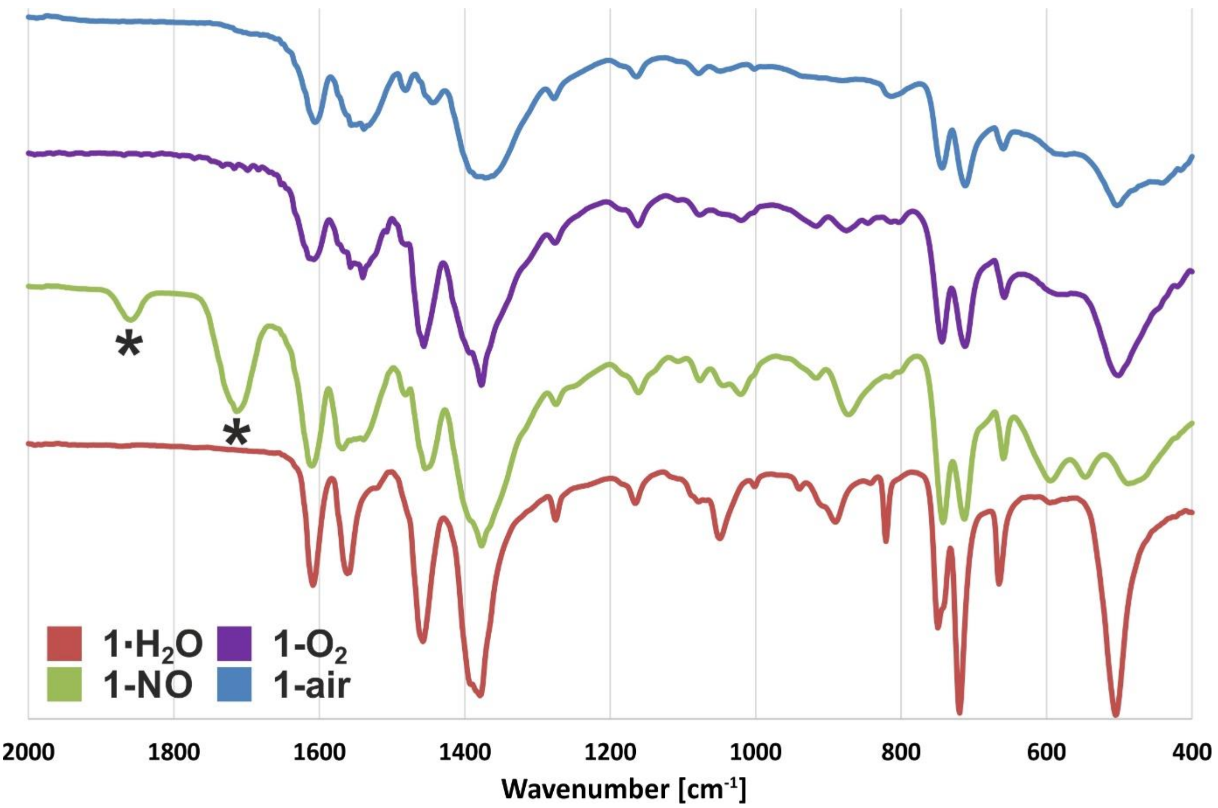

Figure S15. Comparison of the FTIR (ATR) spectra of materials **1·H_2O**, **1-NO**, **1-O_2** and **1-air**. *The signals at 1855 and 1714 cm^{-1}, attributed to vibrations of the Cr-NO group.

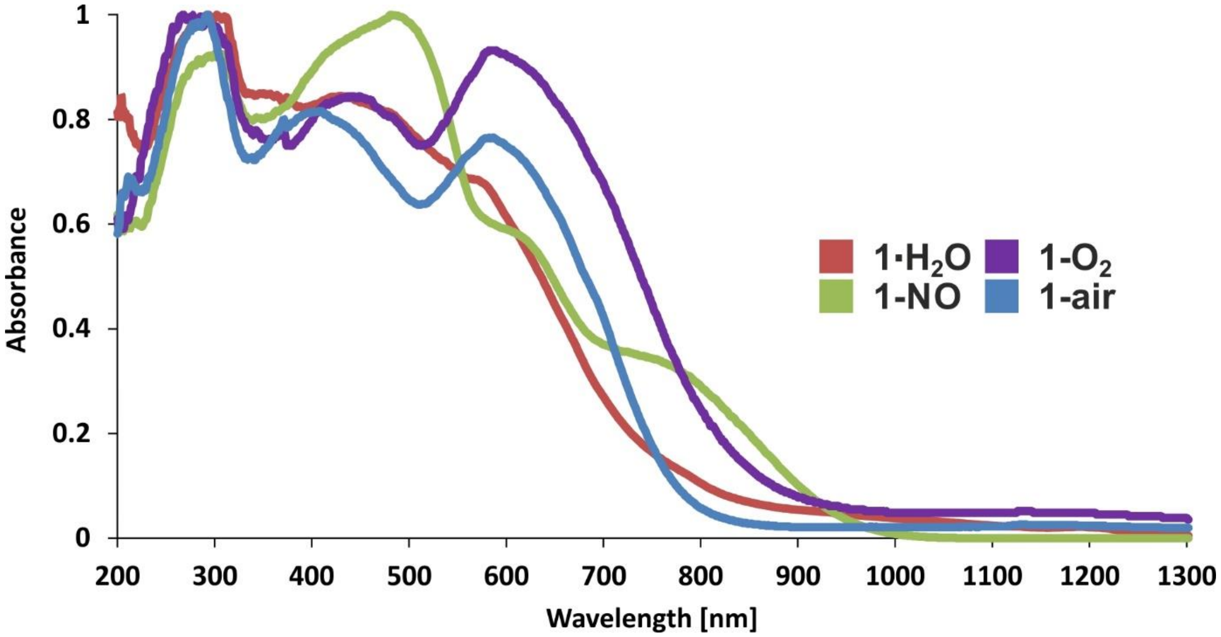

Figure S16. Normalised solid state UV-Vis spectra of **1·H_2O**, **1-NO**, **1-O_2** and **1-air.**

References

1. Zhang, L.; Wang, C.; Zhao, X.; Yu, F.; Yao, F.-F.; Li, J. Three Porous and Robust Metalloporphyrin Frameworks Exhibiting Preferable Gas Storage. *Inorg. Chem. Commun.*, **2015**, *55*, 123–128.
2. Rao, K. P.; Higuchi, M.; Suryachandram, J.; Kitagawa, S. Temperature-Stable Compelled Composite Superhydrophobic Porous Coordination Polymers Achieved via an Unattainable de Novo Synthetic Method. *J. Am. Chem. Soc.*, **2018**, *140* (42), 13786–13792.
3. Dincă, M.; Long, J. R. Strong H_2 Binding and Selective Gas Adsorption within the Microporous Coordination Solid $Mg_3(O_2C\text{-}C_{10}H_6\text{-}CO_2)_3$. *J. Am. Chem. Soc.*, **2005**, *127* (26), 9376–9377.
4. Makal, T. A.; Zhuan, W.; Zhou, H.-C. Realization of Both High Hydrogen Selectivity and Capacity in a Guest Responsive Metal–Organic Framework. *J. Mater. Chem. A*, **2013**, *1*, 13502–13509.
5. Mukherjee, S.; Chen, S.; Bezrukov, A. A.; Mostrom, M.; Terskikh, V. V.; Franz, D.; Wang, S.; Kumar, A.; Chen, M.; Space, B.; Huang Y.; Zaworotko, M. J. Ultramicropore Engineering by Dehydration to Enable Molecular Sieving of H_2 by Calcium Trimesate. *Angew. Chem. Int. Ed.*, **2020**, *59* (37), 16188–16194.
